# Antifungal Compounds Produced by *Colletotrichum gloeosporioides*, an Endophytic Fungus from *Michelia champaca*

**DOI:** 10.3390/molecules191119243

**Published:** 2014-11-21

**Authors:** Vanessa Mara Chapla, Maria Luiza Zeraik, Ioanis Hcristos Leptokarydis, Geraldo Humberto Silva, Vanderlan Silva Bolzani, Maria Claudia M. Young, Ludwig Heinrich Pfenning, Angela Regina Araújo

**Affiliations:** 1 NuBBE - Núcleo de Bioensaios, Biossíntese e Ecofisiologia de Produtos Naturais, Departamento de Química Orgânica, Instituto de Química, UNESP, Universidade Estadual Paulista, Araraquara-SP 14800-900, Brazil; E-Mails: vanessachapla@gmail.com (V.M.C.); marialuizaze@gmail.com (M.L.Z.); ioanlept@gmail.com (I.H.L.); bolzaniv@iq.unesp.br (V.S.B.); 2 Instituto de Ciências Exatas, Universidade Federal de Viçosa, Viçosa, MG, 38810-000, Brazil; E-Mail: silvagh@hotmail.com; 3 Instituto de Botânica, Núcleo de Pesquisa em Fisiologia e Bioquímica, São Paulo-SP, 04301-902, Brazil; E-Mail: marxyoungmc@gmail.com; 4 Departamento de Fitopatologia, Universidade Federal de Lavras, Lavras, MG, 37200-000, Brazil; E-Mail: ludwig@dfp.ufla.br

**Keywords:** *Colletotrichum gloeosporioides*, endophytic fungus, antifungal activity

## Abstract

In this study, eight endophytic fungi were isolated from the leaves, stems and roots of *Michelia champaca*. The isolates were screened and evaluated for their antifungal, anticancer and acetylcholinesterase (AChE) inhibitory activities. All of the extracts exhibited potent activity against two evaluated phytopathogenic fungi. Chemical investigation of EtOAc extracts of the endophytic fungus *Colletotrichum gloeosporioides* resulted in the isolation of one new compound, 2-phenylethyl 1*H*-indol-3-yl-acetate (**1**), and seven known compounds: uracil (**2**), cyclo-(S*-Pro-S*-Tyr) (**3**), cyclo-(S*-Pro-S*-Val) (**4**), 2(2-aminophenyl)acetic acid (**5**), 2(4-hydroxyphenyl)acetic acid (**6**), 4-hydroxy- benzamide (**7**) and 2(2-hydroxyphenyl)acetic acid (**8**). All of the compound structures were elucidated using 1D and 2D NMR and MS analyses. The antifungal and AChE inhibitory activities of compounds **1**–**8** were evaluated* in vitro*. Compound **1** exhibited promising activity against *Cladosporium cladosporioides* and *C. sphaerospermum* that was comparable to that of the positive control nystatin.

## 1. Introduction

The *Colletotrichum* genus is considered a major plant pathogen worldwide. The species *Colletotrichum gloeosporioides* Penz. (teleomorph *Glomerella cingulata*) has been isolated as a plant pathogen [1] and an endophytic fungus [2]. This species has been extensively investigated, particularly for the production of secondary metabolites. Several new antimicrobial metabolites have been isolated from *C. cladosporioides*, including colletotric acid [2], the phytotoxin ferricrocin [3] and gloeosporone [4].

*Michelia champaca* (Magnoliaceae) is native to Asia but is also cultivated in Brazil. This species is used in Brazilian traditional medicine for the treatment of various diseases, including rheumatism, inflammation, fever, cough and fertility regulation. This plant is reported to possess significant antitumor, anti-inflammatory, antimicrobial, antioxidant, antidiabetic, antidiuretic and memory enhancing activities [5]. Secondary metabolites of this plant have been reported, including alkaloids, saponins, tannins, sterols, flavonoids and triterpenoids [6]. Additionally, this plant harbors several endophytes, which produce different classes of metabolites. These observations prompted us to launch a program to isolate novel bioactive metabolites from the cultures of endophytes colonized inside of *M. champaca*.

**Figure 1:**
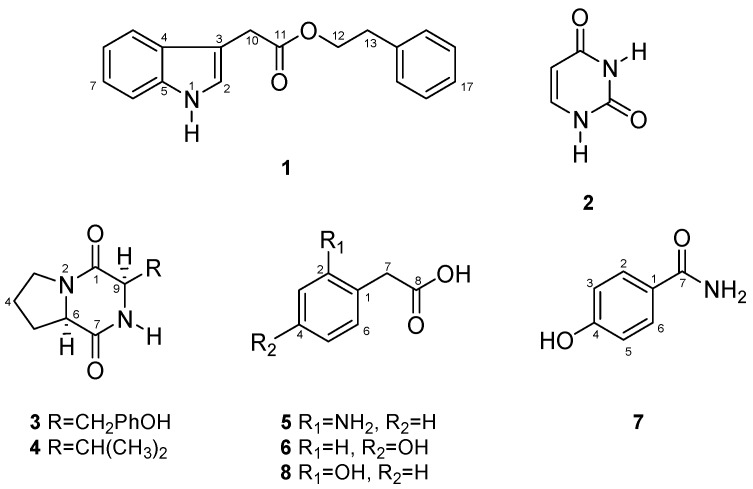
Metabolites produced by *Colletotrichum gloeosporioides*.

Leaves, stems and roots were used for the isolation of endophytic fungi, and eight strains were isolated and evaluated for their biological activity. From the endophytic fungus *C. gloeosporioides*, one new compound 2-phenylethyl 1*H*-indol-3-yl-acetate (**1**) and seven known compounds **2**–**8** (Figure 1) were isolated and identified. Furthermore, the antifungal and anticholinesterase activities of these compounds were evaluated. 

## 2. Results and Discussion

### 2.1. Biological Activities of the Endophytes

Three endophytic fungi were isolated from the stems (Mc-1, Mc-2, and Mc-3), four from the leaves (Mc-4, Mc-5, Mc-6, and Mc-7) and one from the roots (Mc-8) of *M. champaca*. Three different fungi were identified (based on the morphology of the mycelial colony) as *Xylaria* sp.* (*Mc-3), *Phomopsis stipata* (Mc-6) and *C. gloeosporioides* (Mc-7).

The extraction procedure of the eight endophytic fungi isolated from *M. champaca* yielded the following EtOAc crude extracts: Mc-1 (15.6 mg), Mc-2 (7.3 mg), Mc-3 (91.3 mg), Mc-4 (5.2 mg), Mc-5 (8.7 mg), Mc-6 (30.5 mg), Mc-7 (15.0 mg), and Mc-8 (48.5 mg). Screening of the biological activities of these EtOAc crude extracts was performed to select the bioactive fungal strains for subsequent studies.

The extracts were evaluated against the phytopathogenic fungi *C. cladosporioides* and *C. sphaerospermum*, the inhibition of AChE was determined, and the anticancer activities were assessed using *Saccharomyces cerevisiae* (Table 1). The use of *S. cerevisiae* is a rapid and efficient model system to detect anticancer compounds [7].

**Table 1:** Biological activity evaluation of the endophytic fungi isolated from *Michelia champaca*.

Fungus Code	Fungus Identification	Antifungal Activity ^a^		AChE Inhibition ^b^	Anticancer Activity ^c^
		*C.* *sphaerospermum*	*C. cladosporioides*		
Mc-1		++	++	+	+
Mc-2		+	+	++	+
Mc-3	*Xylaria* sp*.*	+++	+++	++	+
Mc-4		+	++	++	+
Mc-5		+	++	++	+
Mc-6	*Phomopsis stipata*	+++	++	++	++
Mc-7	*Colletotrichum gloeosporioides*	+	+++	++	++
Mc-8		+++	+++	+	++

Notes:^ +^ = weak activity; ^++^ = moderate activity; ^+++^ = strong activity; ^a^ Classification is based on the zone diameter (the amount of the sample is 200 µg); ^b^ Classification is based on the zone diameter and retention time (the amount of the sample is 200 µg); ^c^ Classification is based on IC_12_ values (the concentration of the sample is 2000 µg/mL).

The biological activity evaluation indicated all of the fungal extracts exhibited at least moderate antifungal activity against the evaluated phytopathogenic fungi. Additionally, all of the extracts exhibited moderate inhibition of AChE, with the exception of Mc-1, which exhibited weak activity. Only extracts Mc-6, Mc-7 and Mc-8 exhibited moderate anticancer activities. These results were used to select the bioactive fungal strains for subsequent studies.

### 2.2. Molecular Structures and Activities of the Metabolites

NMR and MS analyses of the three identified bioactive fungi indicated that Mc-3 produced a major compound, which was identified as 2-hexylene-3-methylsuccinic acid; this compound has been previously isolated from this fungus, and its antifungal activity has been reported [8]. Mc-6 produced one major compound identified as 3-nitropropionic acid, which has been previously isolated from *Phomopsis* sp*.* and evaluated for potential biological activity against 15 different mycobacteria [9]. Bioactive fungus Mc-7 presented an interesting metabolomic profile with a high number of compounds; this extract was selected for subsequent studies.

Chemical investigation of *C. gloeosporioides* Mc-7 yielded a novel natural product, 2-phenylethyl 1*H*-indol-3-yl-acetate (**1**), together with uracil (**2**) [10], cyclo-(S*-Pro-S*-Tyr) (**3**) [11], biological functions (phytotoxic, antibacterial) were reported for this compound [12], cyclo-(S*-Pro-S*-Val) (**4**) [11], antifungal effects of this compound on two plant pathogens were reported [13], 2(2-aminophenyl)-acetic acid (**5**) [14], 2(4-hydroxyphenyl)acetic acid (**6**) [15], 4-hydroxybenzamide (**7**) [16] and 2(2-hydroxyphenyl)acetic acid (**8**) [14]. The molecular structures of all of the metabolite isolates from *C. gloeosporioides* were elucidated using spectroscopic and chromatographic analyses (2D NMR and LC-MS), which were compared with previously reported values. 

**Figure 2:**
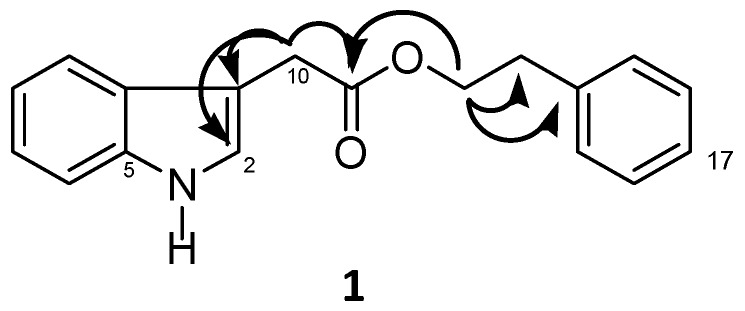
Select *g*HMBC correlations for **1**.

The HR-ESI-MS analysis of compound **1** exhibited an ion at *m/z* 302.1162 [M+Na]^+^ (calcd. for C_18_H_17_NO_2_Na: 302.1151), establishing the molecular formula as C_18_H_17_NO_2_. The ^13^C-NMR data of **1** (Table 2) revealed the presence of 14 aromatic/olefinic carbons, one carbonyl group (*δ_C_* 171.5) and three methylene groups. Analysis of the ^1^H-NMR spectrum revealed *δ*_H_ 6.96 (1H, *dt*, *J* = 8.0 Hz, H-8) and *δ*_H_ 7.08 (1H, *dt*, *J* = 8.0 Hz, H-7) and two doublets at *δ*_H_ 7.42 (1H, *d*, *J* = 8.0 Hz, H-9) and *δ*_H_ 7.36 (1H, *d*, *J* = 8.0 Hz, H-6), which were assigned to an *ortho*-disubstituted aromatic ring. The multiplets at *δ*_H_ 7.14 (2H, *m*, H-15/19), *δ*_H_ 7.25 (2H, *m*, H-16/18) and *δ*_H_ 7.18 (1H, *m*, H-17) were assigned to a monosubstituted aromatic ring. The signals at *δ*_H_ 4.24 (2H, *dt*, *J* = 6.8/1.6 Hz, H-12) and *δ*_H _2.87 (2H, *t*, *J* = 6.8 Hz, H-13) were assigned to an -OCH_2_CH_2_- system; this fragment was established based on ^1^H-^1^H COSY correlations. The H-12 chemical shift (*δ*_H_ 4.24, *δ*_C_ 64.7) and the *g*HMBC correlation to C-11 (*δ*_C_ 171.5) allowed the connection between C-12 to C-11 (Figure 2). The remaining proton at *δ*_H_ 3.70 ppm was assigned as H-10 from *g*HMBC correlation with C-2. Collectively, the planar structure of 2-phenylethyl 1*H*-indol-3-yl-acetate (**1**) was determined. All of the spectra are provided in the Supplementary Material.

All of the compounds **1**–**8** were evaluated for their antifungal activities against two phytopathogenic fungi, *C.* *cladosporioides* and *C. sphaerospermum*, using the TLC diffusion method and nystatin as a positive control (1.0 µg). Compounds **1**, **7** and **8** exhibited high antifungal activities against both fungal strains. Compound **6** also demonstrated high antifungal activity, but only against *C.* *cladosporioides*, and moderate activity against *C. sphaerospermum*. The remaining compounds were inactive. All of the isolated metabolites were evaluated at 100 µg.

The bioactive compounds (**1**, **6**, **7** and **8**) were evaluated at amounts ranging from 1 to 100 μg. Novel natural product **1** exhibited potent antifungal activity at 5 µg, which was similar to that observed for the positive control (nystatin), demonstrating the potential of **1** as an antifungal agent. The remaining evaluated compounds exhibited moderate antifungal activity at 25 μg.

**Table 2:** NMR spectroscopic (500 MHz, DMSO*-d_6_*) data for **1**.

Pos.	1		
	*δ_H_* (*J* in Hz)	*δ_C_*	*g*HMBC
1	10.91 (*s*)	N	C-6
2	7.18 (*m*)	127.0	C-10
3	-	106.9	-
4	-	126.3	-
5	-	136.1	-
6	7.36 (*d*, 8.0)	111.4	C-2/C-8
7	7.08 (*dt*, 8.0/1.5)	121.0	C-5/C-8
8	6.96 (*dt*, 8.0/1.5)	118.5	C-4/C-6
9	7.42 (*d*, 8.0)	118.4	C-5/C-7
10	3.70 (*s*)	30.8	C-2/C-3/C-11
11	-	171.5	-
12	4.24 (*dt*, 6.8/1.2)	64.6	C-11/C-12/C-14
13	2.87 (*t*, 6.8)	34.4	C-12/C-13/C15/C-19
14	-	138.0	-
15	7.14 (*m*)	128.8	C-13/C-15/C-19
16	7.25 (m)	128.3	C-14/C-16/C-18
17	7.18 (*m*)	124.0	-
18	7.25 (*m*)	128.3	C-14/C-16/C-18
19	7.14 (*m*)	128.8	C-13/C-15/C-19

In our bioprospecting program to discover novel anti-Alzheimer’s agents [17], compounds **1**−**8** were evaluated for their AChE inhibitory activity using bioautography. The preliminary TLC assays exhibited moderate activity for all compounds, which were evaluated at 200 μg; galantamine was used as a positive control (1.0 μg).

## 3. Experimental Section

### 3.1. Isolation and Identification of the Endophytic Fungi

Authenticated *M. champaca* was collected in the Chemistry Institute, São Paulo State University (UNESP), Araraquara, São Paulo, Brazil, in April of 2004. A voucher specimen was deposited at the Institute of Botany Herbarium in São Paulo, Brazil.

Endophytic fungi were isolated from the leaves, stems and roots of healthy, adult *M. champaca*, which was subjected to surface sterilization. The leaves, stems and roots were first washed with water and soap and then immersed in 70% aqueous ethanol (EtOH) for 1 min, 1% aqueous sodium hypochlorite solution for 5 min and 70% aqueous EtOH for 1 min. Finally, the vegetal material was immersed in sterile H_2_O for 1 min. The sterilized material was cut into 2 × 2 cm pieces and deposited onto a Petri dish containing PDA (potato dextrose agar) and gentamicin sulfate (100 μg/mL). Single fungal strains were obtained following serial transfers on PDA plates [18]. From the eight isolated endophytic fungi, three were identified as *Xylaria* sp., *P. stipata* and *C. gloeosporioides* by Ludwig H. Pfenning through morphological analyses at the Department of Plant Pathology, Lavras Federal University, Brazil, and deposited in the NuBBE collection under the numbers Mc-3C, Mc-6F, Mc-7C, respectively.

### 3.2. Fungal Growth and Extraction

The endophytic fungi isolated from *M. champaca* were each inoculated into two Erlenmeyer flasks (500 mL), each containing PDB medium (250 mL). The medium was autoclaved at 121 °C for 15 min. After cooling, the medium was inoculated with the endophytes and incubated at 25 °C on rotary shakers at 150 rpm for 28 days. The flask-accumulated mycelial biomass was separated from the aqueous medium by filtration, and the filtrate was subjected to liquid-liquid partition with ethyl acetate (EtOAc; 3 × 300 mL). The EtOAc fraction was evaporated *in vacuo*, resulting in the EtOAc crude extract. *C. gloeosporioides* was cultured on a larger scale and inoculated into six Erlenmeyer flasks (4 L), each containing PDB medium (3.0 L), and incubated at 25 °C on rotary shakers at 150 rpm for 28 days. The mycelial biomass was separated and fractionated as previously described.

### 3.3. Isolation and Identification of the Active Metabolites

The EtOAc crude extract (1.6 g) obtained from large-scale cultures was fractionated by column chromatography (CC) using C_18_ silica gel (Merck, Darmstadt, Germany) as the stationary phase and eluted with a H_2_O:MeOH gradient (5%–100% MeOH) containing 5% of acetic acid, affording 11 fractions (Fr1–Fr11). From these fractions, eight compounds were identified, including one new natural product. 

These compounds were structurally identified by HRMS and NMR analyses. ^1^H-NMR (500 MHz), ^13^C-NMR (125 MHz), *g*HMBC, *g*HMQC and *g*COSY experiments were conducted on a Varian DRX-500 spectrometer using the non-deuterated residual solvent signal as a reference. Mass spectra were acquired on a Bruker ultrOTOF-Q-ESI-TOF mass spectrometer using MeOH or MeOH/H_2_O as the eluent (cone voltage: 25 V).

Fraction Fr1 (235.0 mg) was separated on a Varian ProStar (SD-1 Solvent Delivery Module) preparative HPLC coupled with a Varian ProStar 320 UV/Vis detector system using a C-18 (Phenomenex, Luna, 250.0 mm × 21.2 mm, 10 μm) preparative column. A H_2_O:5% AcOH in MeOH (83:17 v/v, 20 mL/min, *λ*_max_ = 320 nm) mixture was used as the eluent, yielding pure compounds **2** (16.7 mg), **3** (28.5 mg) and **4** (53.2 mg). Fraction Fr2 (379.2 mg) was subjected to preparative HPLC using H_2_O:5% AcOH in MeOH (80:20 v/v, 20 mL/min, *λ*_max_ = 320 nm) as the eluent, yielding pure compounds **5** (16.0 mg), **6** (20.5 mg), **7** (18.5 mg) and **8** (30.2 mg). Novel natural product **1** (51.7 mg) was obtained from Fr9.

All compounds were analyzed by analytical HPLC performed on a Varian ProStar (240 Solvent Delivery Module and 410 AutoSampler) coupled with a Varian ProStar 330 photodiode array ultraviolet (PDA-UV) detector system using a C-18 column (Phenomenex, Luna, 250.0 mm × 4.6 mm, 5 μm).

### 3.4. Biological Activity

#### 3.4.1. Antifungal Activity by Bioautography

The extracts and isolated compounds were evaluated against the fungal pathogens *Cladosporium cladosporioides* (Fresen) Vries SPC 140 and *C. sphaerospermum* (Perzig) SPC 491. Nystatin was used as a positive control at 1.0 μg. The samples were applied on precoated silica gel TLC plates using a solution (10 μL) that contained 200 μg of the crude extract and 100.0, 50.0, 25.0, 10.0, 5.00 and 1.00 μg of the pure compounds. Following development with CHCl_3_:CH_3_OH (9:1), the plates were sprayed with the fungal suspension and incubated at 25 °C for 2–3 days. The antifungal activities were detected as clear zones on the fungal background [19].

#### 3.4.2. Acetylcholinesterase (AChE) Inhibitory Activity

The AChE inhibitory activities of the extracts and pure compounds were determined using a TLC bioautographic assay as previously described [20]. Galantamine (1.0 μg) was used as a positive control. Compounds **1**–**8** were applied at 100 to 1.0 μg on TLC plates, and the extracts were applied at 200 μg. The compounds and extracts were developed with *n*-hexane‒EtOAc (9:1, v/v) and subsequent drying. The plates were sprayed with an enzyme solution containing electric eel AChE type V (Sigma-Aldrich, St. Louis, MO, USA; 6.66 U/mL), thoroughly dried and incubated at 37 °C for 20 min (under a humid atmosphere). Enzymatic activity was detected by spraying with a solution of 0.25% 1-naphthyl acetate in EtOH with a 0.25% aqueous solution of Fast Blue B salt. Potential AChE inhibitors appeared as clear zones on a purple background [20].

#### 3.4.3. Anticancer Activity

The anticancer activities of the extracts and pure compounds were investigated using DNA repair or recombination-deficient mutants of the yeast *S. cerevisiae* for screening. This bioassay is a model for the evaluation of anticancer activity based on the use of genetically modified yeast strains [21]. All of the extracts were applied at 2 mg/mL (100 μL) to wells containing culture medium with different strains [52Y Rad (0.05–0.1), Rad+ (0.05) and 321 Rad (0.05 to 0.1)] and then incubated for 48 h. The reference compounds camptothecin (4 and 5 μg/mL for RAD^+^ and 52Y Rad, respectively) and streptonigrin (200 μg/mL for 321 Rad) were used. The results are reported as an IC_12_ value, which represents the concentration required to produce a 12-mm inhibition zone diameter around a 100 μL well containing the yeast strain in question. An extract is considered active if it demonstrated selective activity against one or more repair-deficient yeast strains and exhibits an IC_12_ of less than 2000.

## 4. Conclusions

These results suggest the immense potential of endophytic fungi to produce structurally diverse and bioactive chemical scaffolds with both commercial and pharmaceutical significance. The potent antifungal activity of novel natural product **1** suggests that *C. gloeosporioides* plays an important ecological role, protecting *M. champaca* against phytopathogens. Furthermore, eight endophytic fungi were isolated from this traditional herbal medicine and exhibited significant biological activities, demonstrating the pharmacological proprieties of endophytes.

## Supplementary Materials

Supplementary materials can be accessed at: http://www.mdpi.com/1420-3049/19/11/19243/s1.

## Supplementary Files

Supplementary File 1
