# Supplementary material for: Antifungal Compounds Produced by Colletotrichum gloeosporioides, an Endophytic Fungus from Michelia champaca
Source: Molecules. 2014 Nov 21;19(11):19243–52. doi: 10.3390/molecules191119243 (PMC6271623; doi:10.3390/molecules191119243)

Supplementary Materials

Figure S1. ^{1}H-NMR spectrum for compound **1**.

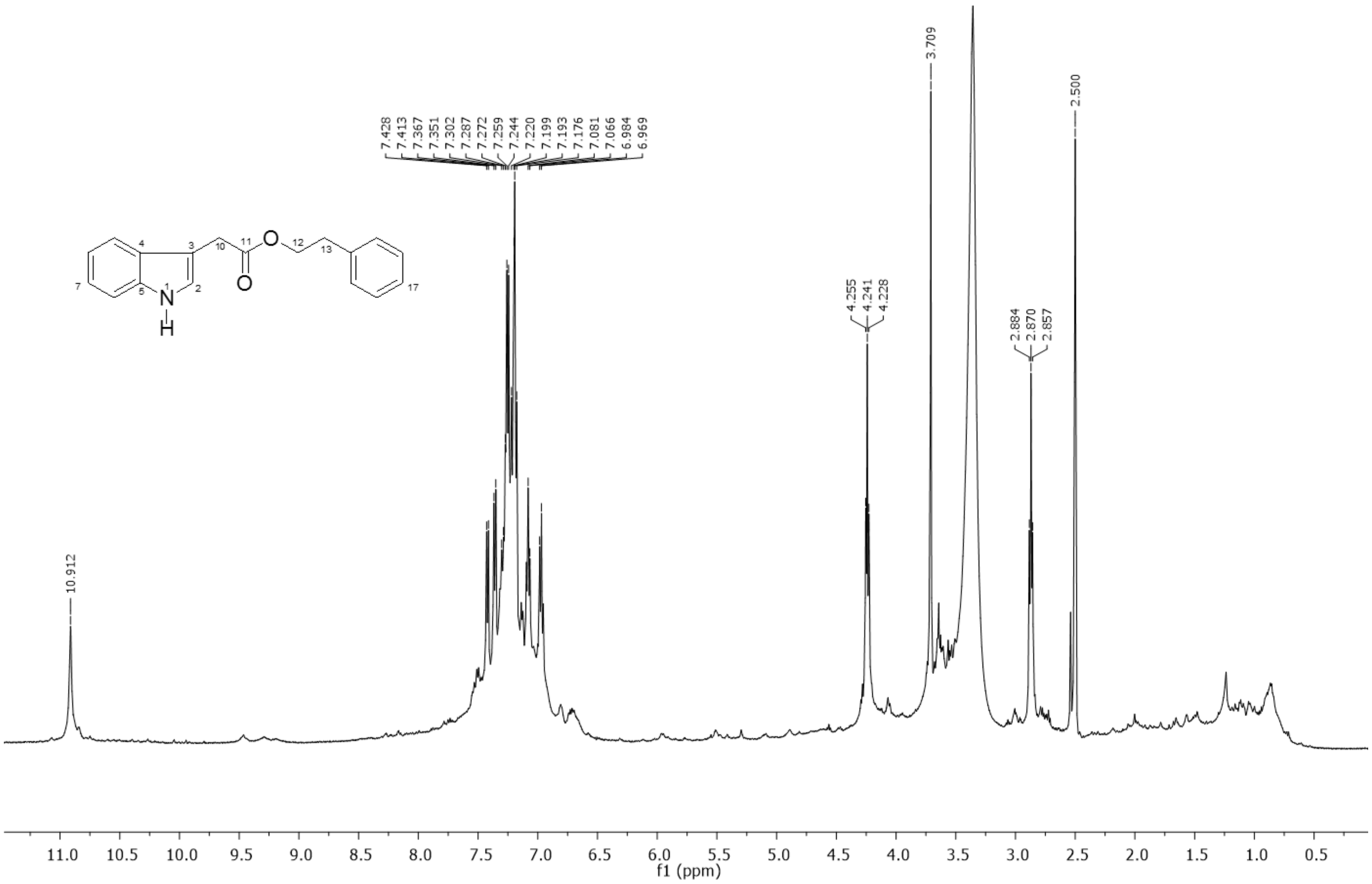

Figure S2. ^{13}C-NMR spectrum for compound **1**.

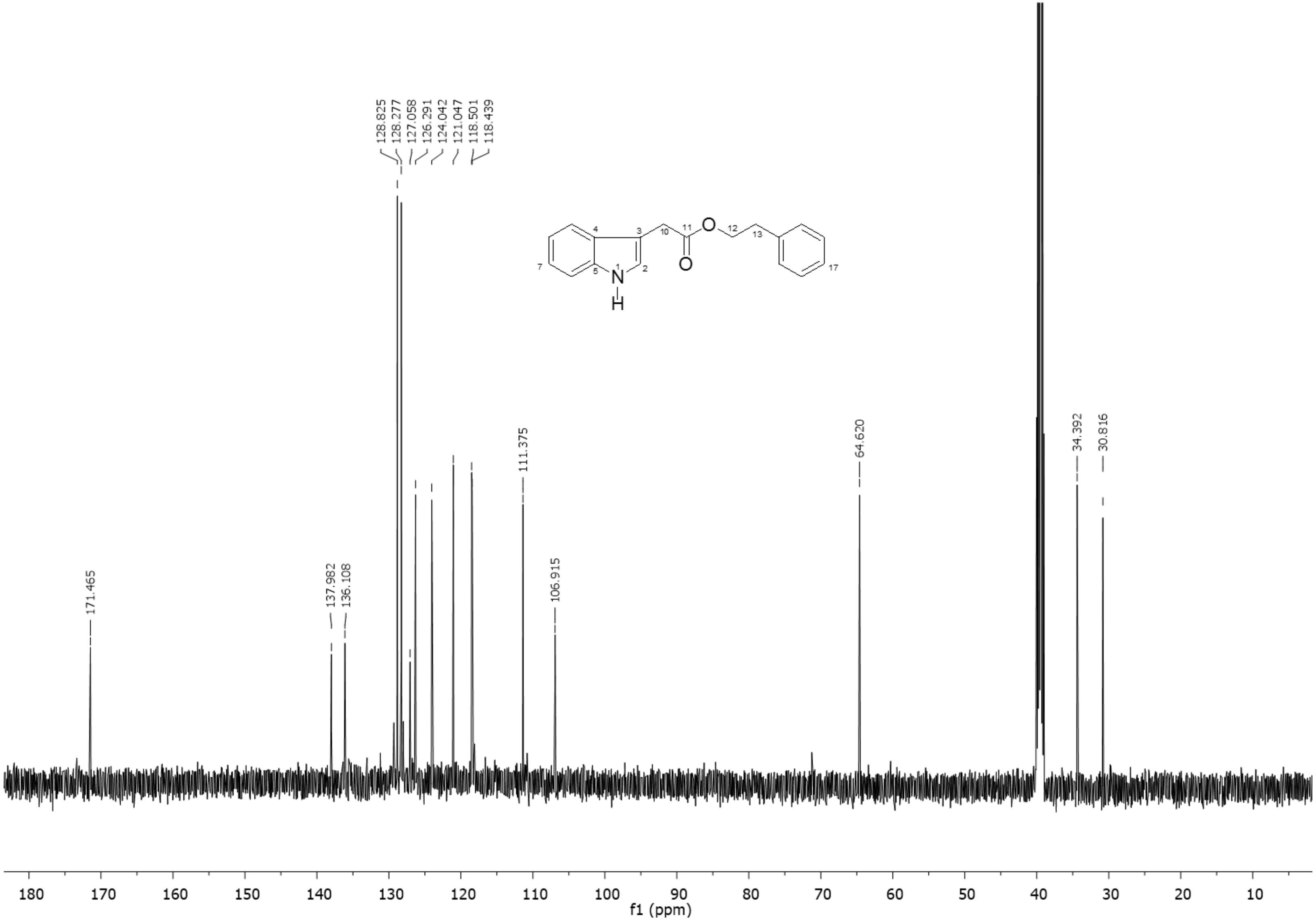

Figure S3. HMQC spectrum for compound **1**.

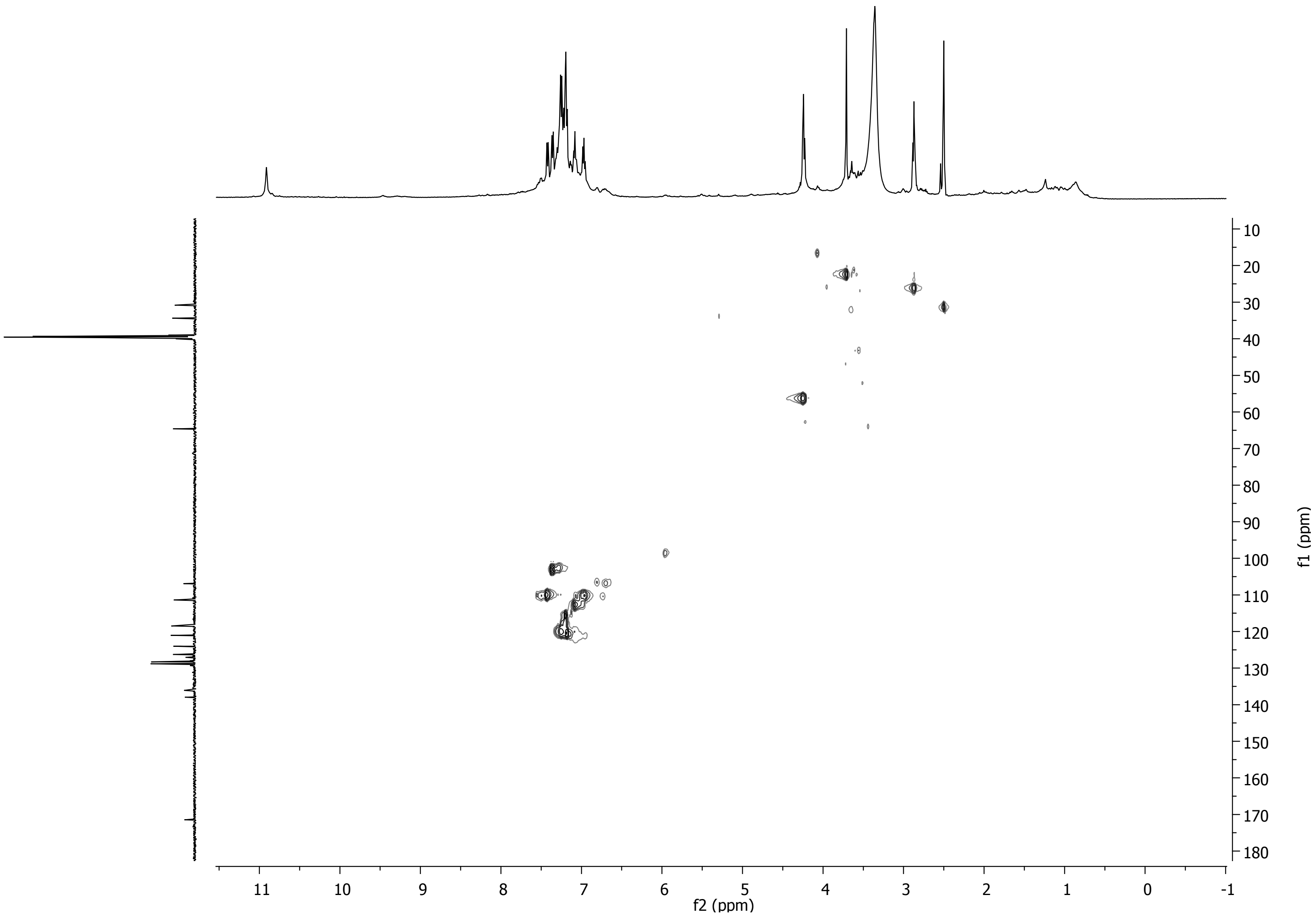

Figure S4. HMBC spectrum for compound **1**.

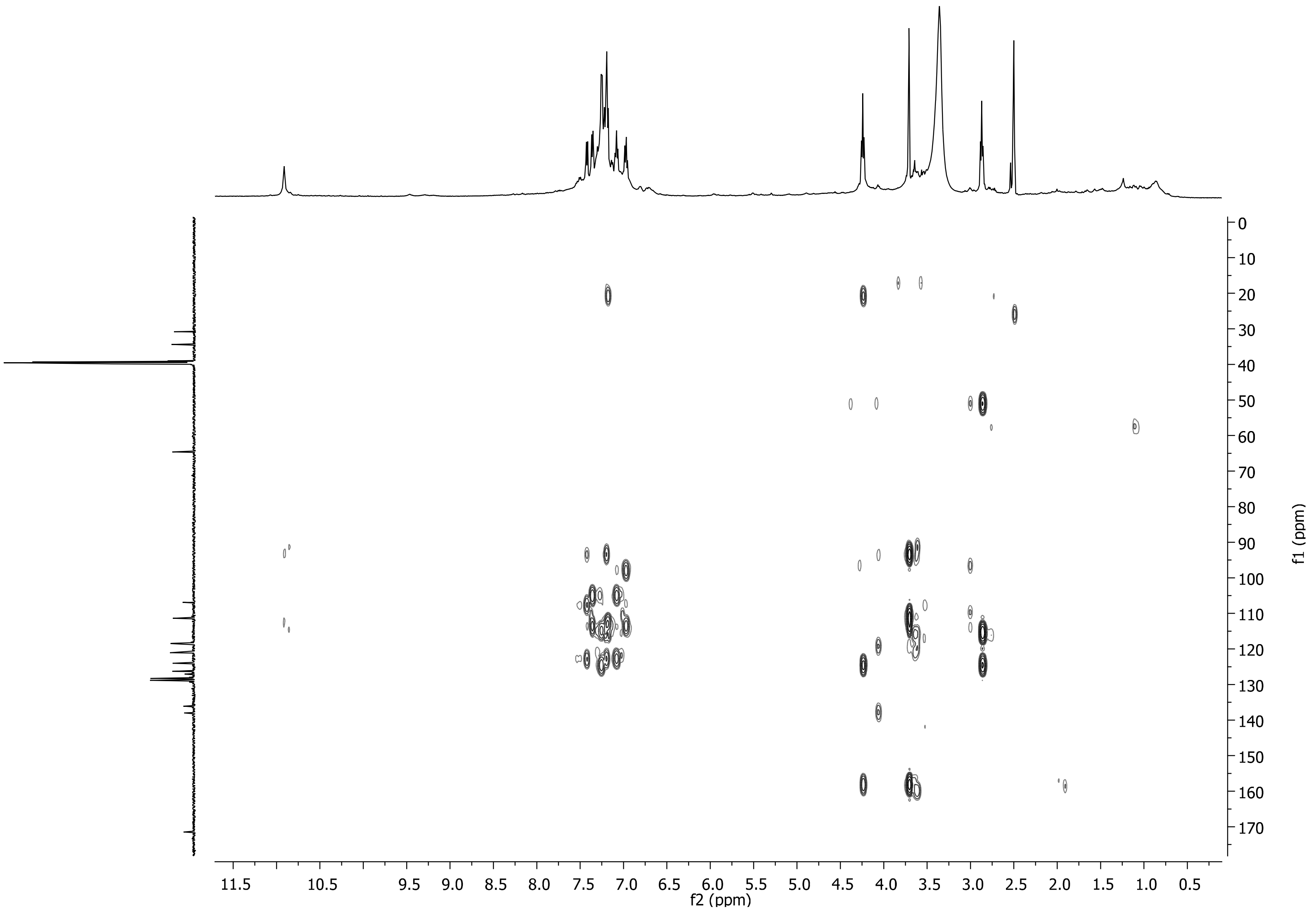

Figure S5. COSY spectrum for compound **1**.

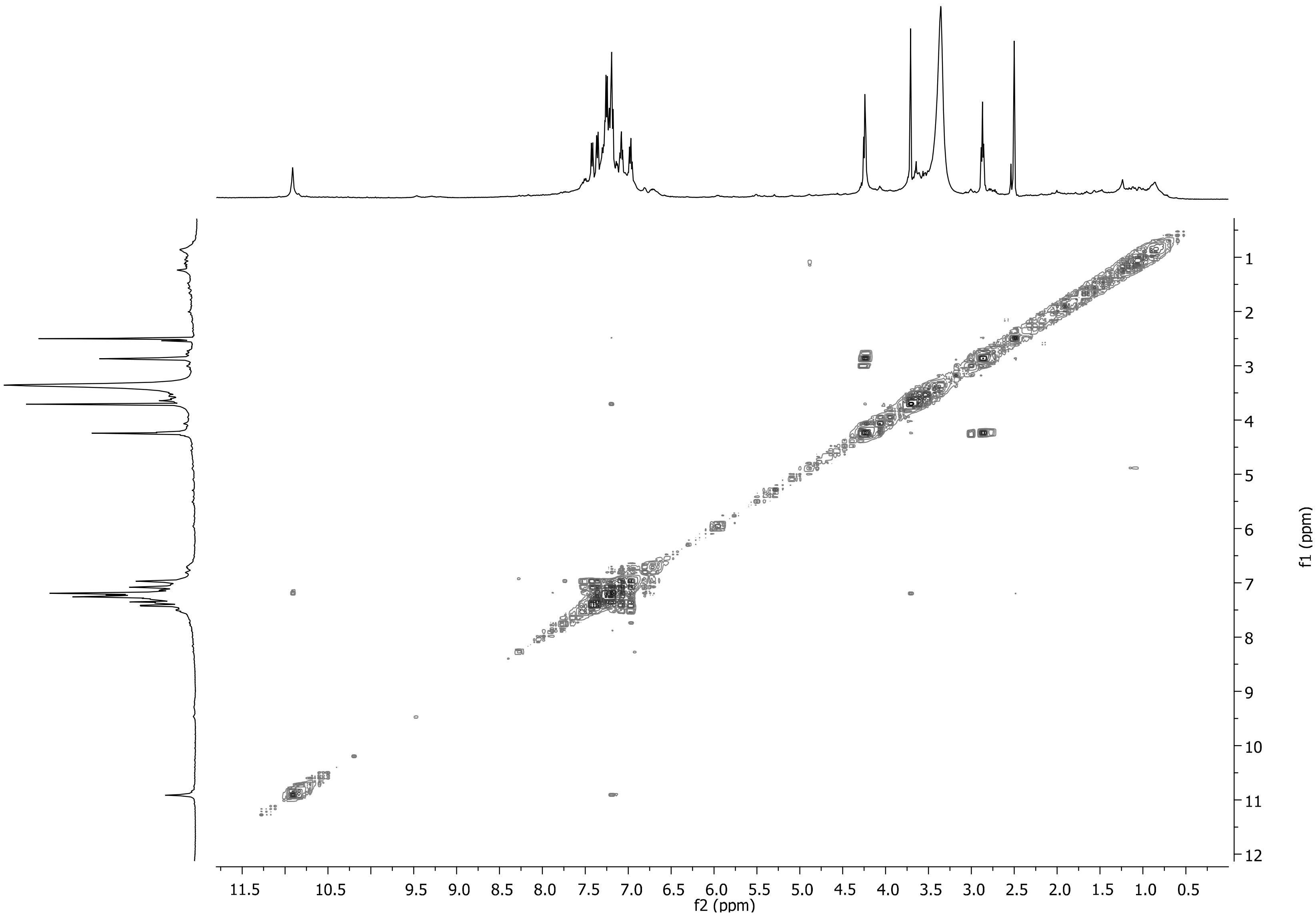

Figure S6. 1D-NOESY spectra for compound **1**.

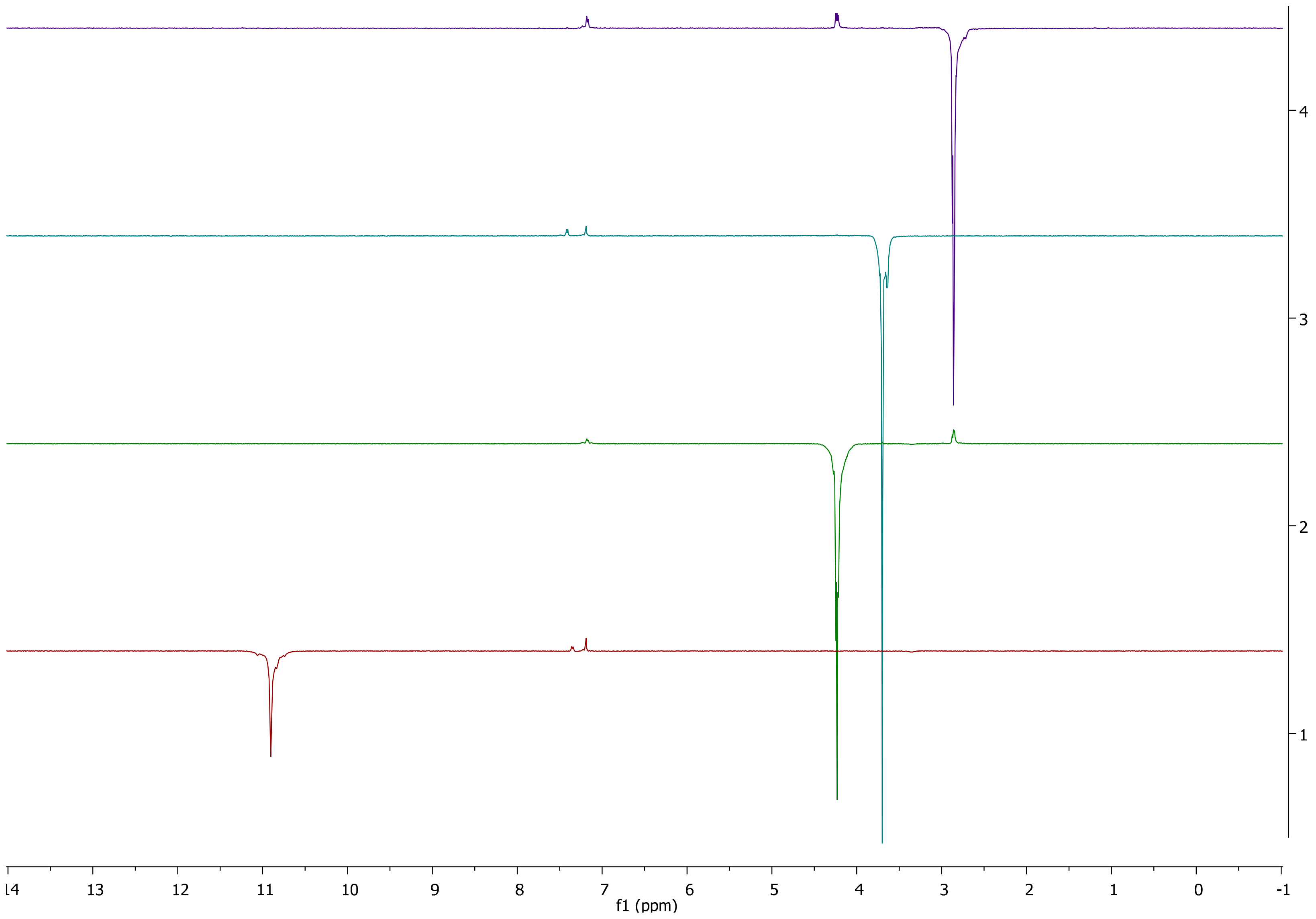

Figure S7. Mass spectrum for compound **1**.

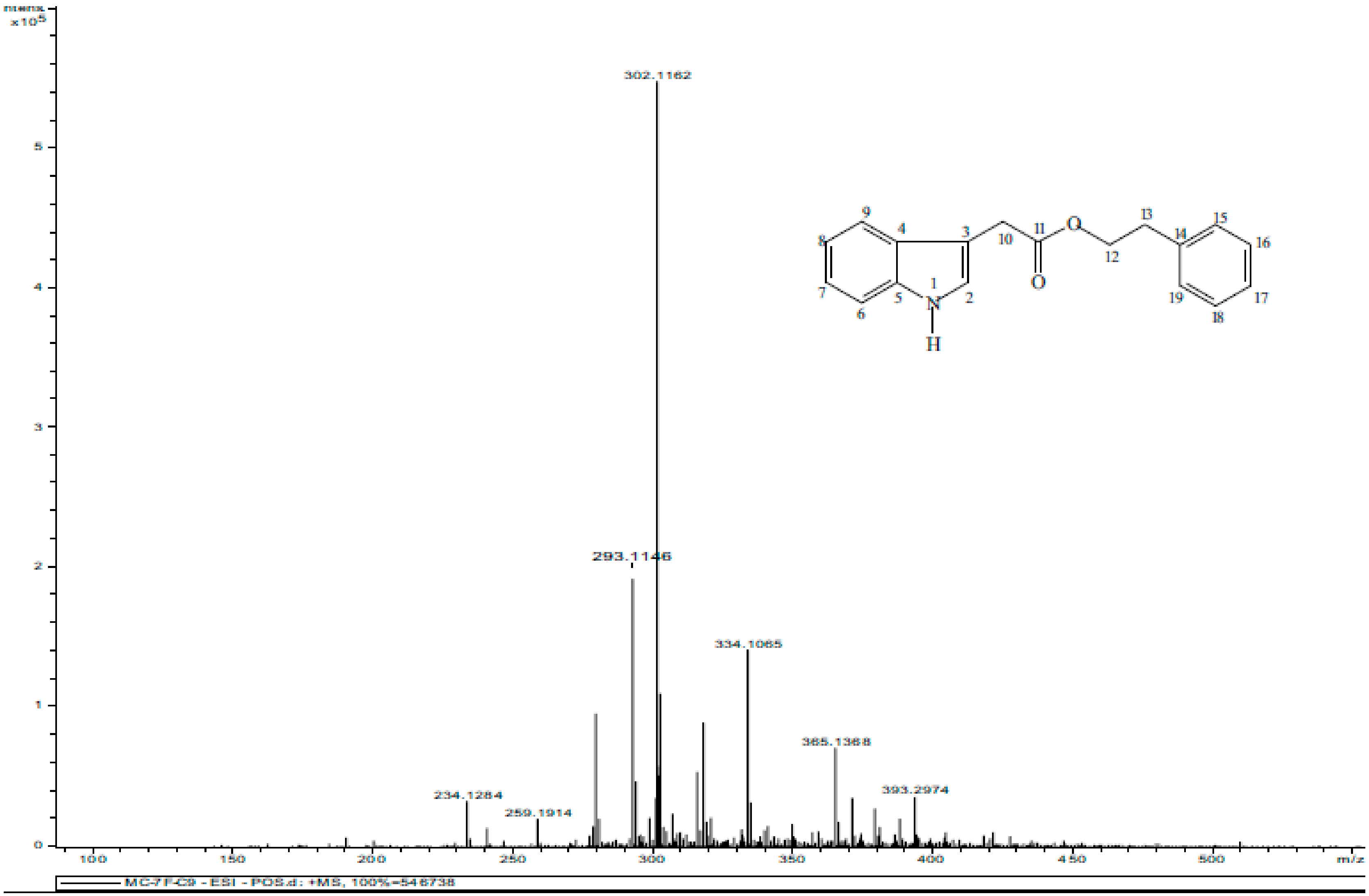

Supplement: Supplementary File 1 [file molecules-19-19243-s001.pdf]
